# Endoscopic management of hemorrhagic pancreatic fluid collections: A propensity‐matched analysis

**DOI:** 10.1002/deo2.195

**Published:** 2022-12-08

**Authors:** Rishi Pawa, Robert Dorrell, Greg Russell, Madison Nguyen, Clancy Clark, Girish Mishra, Swati Pawa

**Affiliations:** ^1^ Department of Medicine Wake Forest University School of Medicine Winston‐Salem North Carolina USA; ^2^ Department of Biostatistics and Data Science Wake Forest University School of Medicine Winston‐Salem North Carolina USA; ^3^ Department of Medicine Virginia Commonwealth University Richmond Virginia USA; ^4^ Department of General Surgery Wake Forest University School of Medicine Winston‐Salem North Carolina USA

**Keywords:** drainage, endoscopic ultrasonography, hemorrhagic pancreatitis, pseudoaneurysm, self‐expandable metal stent

## Abstract

**Objectives:**

Hemorrhagic pancreatic fluid collections (hPFC) are a complication of pancreatitis with an unknown influence on prognosis. Advancements in endoscopic management of PFC have improved results over their surgical and percutaneous alternatives. We performed a propensity‐matched analysis comparing clinical outcomes in hemorrhagic and non‐hemorrhagic PFC (nhPFC).

**Methods:**

From November 2015 to November 2021, a retrospective comparative cohort analysis was performed comparing clinical outcomes for patients with hPFC and nhPFC managed with lumen‐apposing metal stents. Propensity score matching was used to balance the two subgroups. Wilcoxon two‐sample tests were used to compare continuous variables and Fisher's exact test was used to compare categorical variables. Kaplan‐Meier method was used to estimate overall survival.

**Results:**

Fifteen patients with hPFC were matched with 30 nhPFC patients. Technical and clinical success was similar in both groups. The median length of hospitalization was 6 days in the hPFC group and 3 days in the nhPFC group (*p* = 0.23); however, more hPFC patients required intensive care unit admission post‐procedure (33.3% vs. 16.7%, *p* = 0.26). Patients with hPFC were more likely to be readmitted to the hospital within 30 days (33.3% vs. 6.7%, *p* = 0.032). Mortality at 3 months (13% vs 3%, *p* = 0.25) and 6 months (27% vs. 7%, *p* = 0.09) was higher in the hPFC cohort. The 1‐year survival estimate was 73.3% (standard error = 11.4) in the hPFC group and 88.9% (6.1) in the nhPFC group (*p* = 0.16).

**Conclusions:**

Patients with hPFC are more likely to be readmitted to the hospital within 30 days and have worse clinical outcomes.

## INTRODUCTION

Pancreatic fluid collections (PFC) result from acute and chronic pancreatitis during which inflammation compromises gland integrity causing pancreatic juices to leak into the surrounding tissues. These collections are associated with various complications including infection, biliary obstruction, gastric outlet obstruction, hemorrhage, or rupture.^1^, ^2^, ^3^ The presence of hemorrhage is a frequently overlooked complication of PFC. Intracystic hemorrhage typically occurs due to a bleeding pseudoaneurysm, diffuse venous bleeding, or pancreatic necrosis involving luminal vessels.^4^, ^5^ Most existing literature on hemorrhagic PFC (hPFC) has been limited to case reports; therefore, its clinical significance has been largely unstudied. We hypothesize that hPFC likely have a worse outcome than non‐hemorrhagic PFC (nhPFC) and aim to present our experience managing these collections endoscopically.

## MATERIALS AND METHODS

Between November 2015 and November 2021, a retrospective analysis of a prospectively maintained database was performed on all patients who underwent endoscopic ultrasound‐guided drainage of PFC using lumen‐apposing metal stents (LAMS). Institutional Review Board approval was obtained (IRB 00035936). Endoscopic drainage was performed for patients with symptomatic PFC. These symptoms included abdominal pain, gastric outlet obstruction, biliary obstruction, early satiety, and organ failure. All patients who underwent drainage of PFC using LAMS were classified into two groups differentiated by the presence or absence of hemorrhage identified on cross‐sectional imaging (diagnosed as a collection with a density of 50–100 Houndsfield units) and/or endoscopic ultrasound.^6^ Propensity score matching was used to balance the hPFC and nhPFC groups in a 1:2 ratio (hemorrhagic and non‐hemorrhagic, respectively) based on patient characteristics (age, gender, body mass index, etiology, and age‐adjusted Charlson comorbidity index) and collection characteristics (type and size of collection and presence of infection).^7^ Inclusion criteria for the study comprised patients greater than or equal to 18 years of age who underwent endoscopic drainage of PFC with at least a 6‐month follow‐up duration. Exclusion criteria included patients less than 18 years of age, postoperative collections, presence of malignancy on imaging, an international normalized ratio greater than 1.5, and a platelet count of less than 50,000.

### Procedure details

**FIGURE 1:**
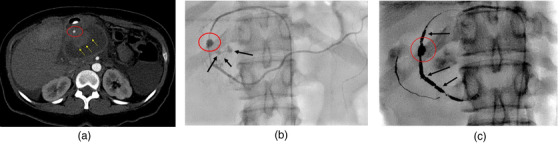
Computed tomography (CT) angiogram with angioembolization of pseudoaneurysm involving gastroduodenal artery. (a) CT angiogram showing gastroduodenal artery pseudoaneurysm (red circle) with surrounding hematoma (yellow arrows). (b) Angiogram showing pseudoaneurysm (red circle) with active extravasation into the pseudocyst (black arrows). (c) Coils packing the pseudoaneurysm (red circle) and gastroduodenal artery (black arrows)

**FIGURE 2:**
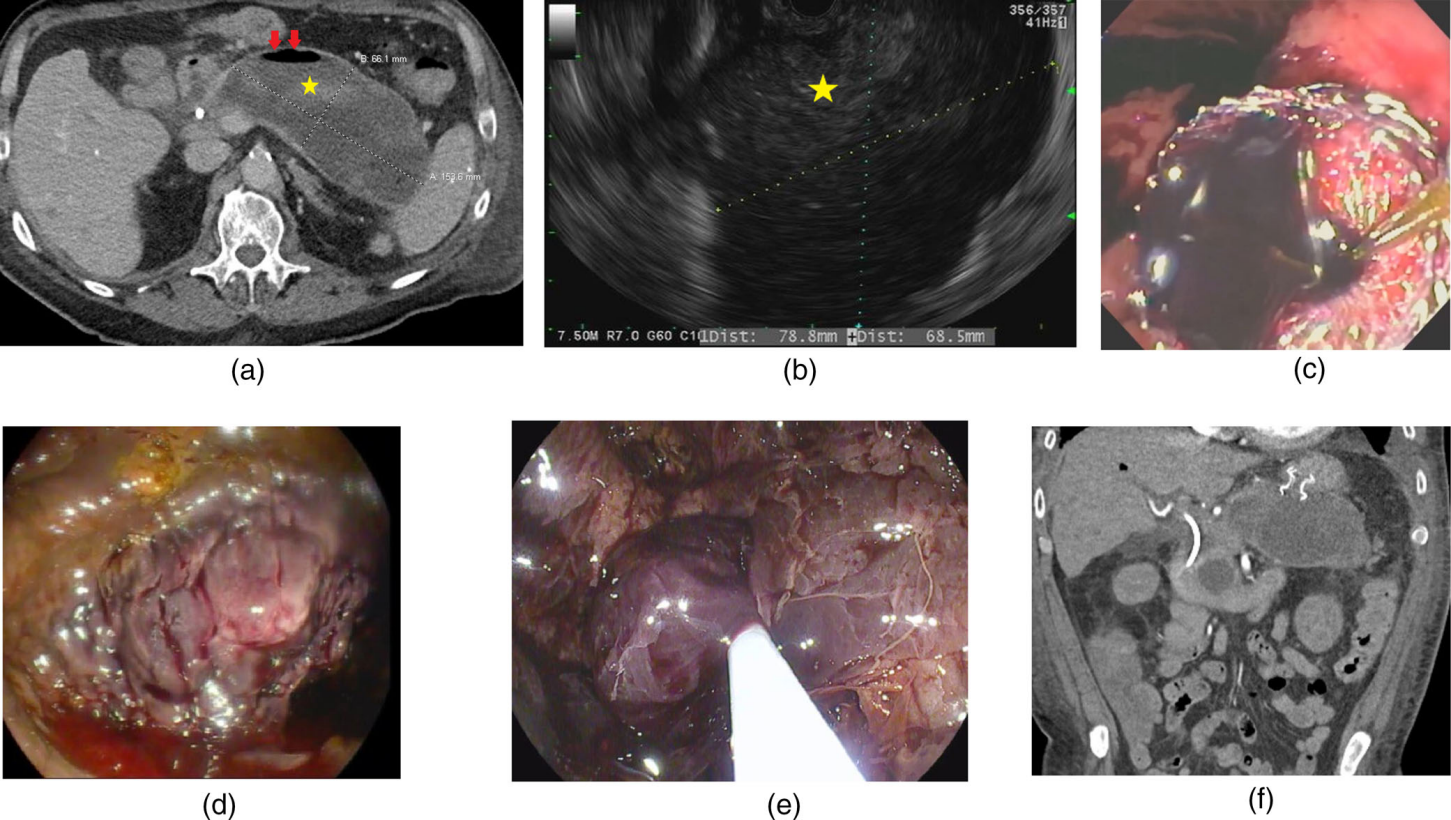
Endoscopic ultrasound‐guided drainage of hemorrhagic pancreatic fluid collection using a lumen‐apposing metal stent. (a) Computed tomography (CT) abdomen showing a large pancreatic fluid collection with hyperdense contents (asterisk) and air (red arrow) suggestive of infected hemorrhagic pancreatic fluid collection. (b) Endoscopic ultrasound showing a large pancreatic fluid collection with echogenic material (asterisk) suggestive of hemorrhage. (c) Endoscopic imaging showing blood draining from the cyst cavity after lumen apposing metal stent placement. (d) Endoscopic image showing blood clots inside the cyst cavity. (e) Endoscopic image showing removal of blood clots using a snare. (f) CT abdomen (coronal view) showing the appropriate position of lumen‐apposing metal stents (LAMS) inside the cyst cavity

All patients with hPFC underwent computed tomography (CT) angiogram to assess the presence of pseudoaneurysm prior to endoscopic drainage. Selective angioembolization was performed if a pseudoaneurysm was identified (Figure 1) and PFC drainage was delayed for 48 hours after embolization. Endoscopic drainage was performed under general anesthesia and intravenous antibiotics were administered at the start of the procedure. A curvilinear echoendoscope (GF‐UCT180; Olympus, Tokyo, Japan) was then advanced into the stomach and duodenum to visualize the collection. Doppler was used to identify intervening luminal vessels and identify a safe deployment tract for the LAMS (Axios; Boston Scientific, Marlborough, MA). A 15mm X 10mm LAMS was used in all patients with walled‐off pancreatic necrosis and a 10 × 10 mm LAMS was used in all patients with pseudocysts. Following the successful deployment of the LAMS, the stent was dilated to its diameter using a through‐the‐scope dilating balloon. Necrotic collections were debrided with rat tooth forceps, snares, or baskets at the discretion of the endoscopist (Figure 2). Serial imaging was performed at regular intervals (1 week, 3–4 weeks, 6 weeks after initial stent placement, and 1‐week following every necrosectomy) to determine the resolution of the collection and the need for reintervention. The presence or absence of disconnected pancreatic duct syndrome was evaluated by either magnetic resonance cholangiopancreatography or endoscopic retrograde pancreatography. The timing and interval of each necrosectomy were based on the patient's symptoms and imaging findings concerning persistent necrosis in the cyst cavity. The LAMS was removed when the collection size was less than or equal to 2 cm on follow‐up imaging with improvement in symptoms. Patients who failed endoscopic management were evaluated for percutaneous drainage or surgical step‐up.

### Endpoints

Technical success was defined as the successful deployment of LAMS into the cyst cavity. Clinical success was defined as a decrease in the size of PFC to less than or equal to 2 cm at the time of LAMS removal with a resolution of symptoms. Recurrence of PFC was defined as increasing the size of the prior drained collection (greater than 2 cm) on subsequent imaging. Follow‐up duration was measured from the time of LAMS placement to the most recent clinic visit with a gastroenterologist. Adverse events were recorded and graded with the American Society of Gastrointestinal Endoscopy Lexicon criteria.^8^

Secondary endpoints included length of hospitalization, length of intensive care unit (ICU) stay, in‐hospital mortality, mortality at 3 and 6 months, 30‐day hospital readmission rate, and overall survival. The criteria for admission or readmission following discharge were the need for medical care that could not be provided as an outpatient. This included patients with symptoms related to their collection including but not limited to intractable abdominal pain requiring intravenous analgesia, evidence of hemodynamic instability, concern for infection requiring intravenous antibiotics, sepsis, single organ or multiorgan failure, and intractable nausea and vomiting secondary to gastric outlet obstruction. Patients were discharged after the procedure if none of the above criteria were present. ICU stay was defined as a patient admitted to the hospital under the care of an intensive care specialist with options for mechanical ventilation or vasopressor support.

### Statistical analysis

Descriptive statistics were used to compare the two groups (hemorrhagic and non‐hemorrhagic). Propensity score matching employing a greedy‐matching algorithm and no replacement was used to balance the hemorrhagic and non‐hemorrhagic patients in a 1:2 fashion. The balanced samples were compared using Fisher's exact test for categorical measures and Wilcoxon two‐sample tests for continuous outcomes. The Chi‐square approximation of the log‐rank test was used to assess differences between groups in survival outcomes. The Kaplan‐Meier method was used to estimate overall survival in the propensity score‐matched cohorts. Statistical analysis system (version 9.4; Cary, NC, USA) was used for all analyses.

## RESULTS

**FIGURE 3:**
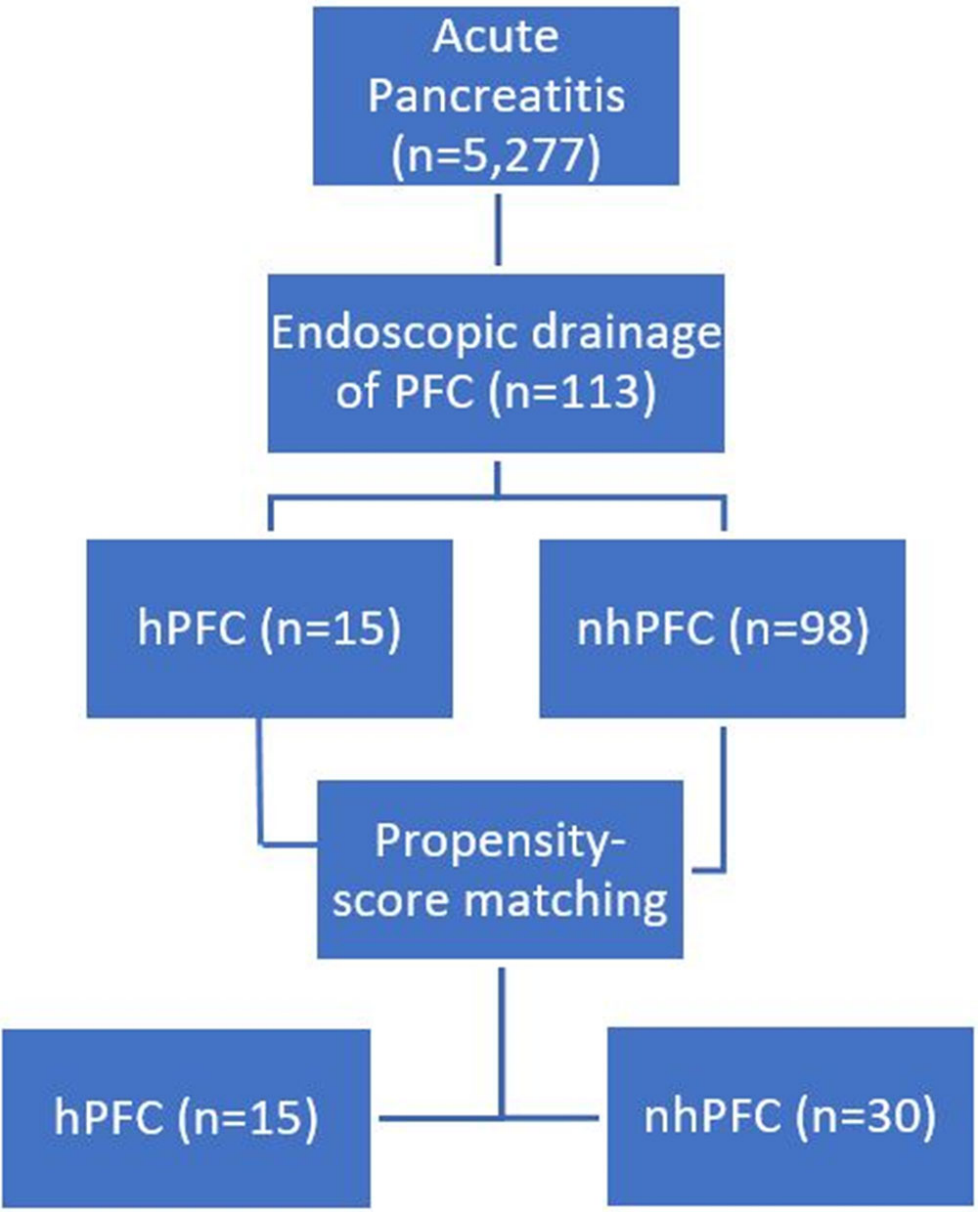
Study flowchart

There were 5277 patients diagnosed with acute pancreatitis during the study period. Of the 113 patients who underwent endoscopic management of symptomatic PFC using LAMS, 15 (12.6%) were found to have hemorrhagic collections. A pseudoaneurysm was diagnosed on a CT angiogram in two patients who underwent interventional radiology‐guided angioembolization prior to endoscopic drainage. By propensity score matching at a ratio of 1:2, the hPFC patients were matched with 30 patients from the nhPFC group (98 patients) resulting in a total of 45 patients (Figure 3). Table 1a, 1b summarizes the variables included in the propensity score matching, pre‐, and post‐matching, presenting the standardized mean difference. Following matching and using an standardized mean difference of 0.1 as the reference value, there was no statistically significant difference between the two groups.

**TABLE 1a:** Parameters included in propensity score matched analysis, pre‐matching

**Patient characteristics**	**Hemorrhagic PFC (*n* = 15)**	**Non‐hemorrhagic PFC (*n* = 98)**	**SMD**
Age, median (IQR)	61 (43, 67)	54 (45, 63)	0.18
Gender (female), *n*, %	6, 40%	35, 36%	0.09
Body mass index, median (IQR)	25.7 (23.4, 27.8)	27.5 (23.9, 33.1)	0.40
Etiology, *n*, %			
Alcohol	5, 33%	40, 41%	0.18
Gallstone	6, 40%	33, 34%	0.15
Idiopathic	3, 20%	18, 18%	0.06
Hypertriglyceridemia	1, 7%	3, 3%	0.45
Medication‐induced	0, 0%	4, 4%	>1
Charlson comorbidity index, median (IQR)	4 (1, 4)	3 (1, 4)	0.31
Presence of infection, *n*, %	8, 53%	31, 32%	0.50
Area of collection (cm^2^), median (IQR)	120 (55, 153)	79 (45, 156)	0.002
Pseudocyst, *n*, %	1, 7%	29, 30%	0.62
Walled‐off necrosis, *n*, %	14, 93%	69, 70%	0.96

Abbreviations: IQR, interquartile range; PFC, pancreatic fluid collections; SMD, standardized mean difference.

**TABLE 1b:** Parameters included in propensity score matched analysis, post‐matching

**Patient characteristics**	**Hemorrhagic PFC (*n* = 15)**	**Non‐hemorrhagic PFC (*n* = 30)**	**SMD**
Age, median (IQR)	61 (43, 67)	59 (46, 63)	0.04
Gender (female), *n*, %	6, 40%	11, 37%	0.07
Body mass index, median (IQR)	25.7 (23.4, 27.8)	25.8 (22.9, 28.0)	0.07
Etiology, *n*, %			
Alcohol	5, 33%	9, 30%	0.07
Gallstone	6, 40%	12, 40%	0
Idiopathic	3, 20%	7, 23%	0.09
Hypertriglyceridemia	1, 7%	2, 7%	0
Charlson comorbidity index, median (IQR)	4 (1, 4)	4 (1, 4)	0.09
Presence of infection, *n*, %	8, 53%	15, 50%	0.07
Area of collection (cm^2^), median (IQR)	120 (55, 153)	94 (55, 124)	0.099
Pseudocyst, *n*, %	1, 7%	2, 7%	0
Walled‐off necrosis, *n*, %	14, 93%	28, 93%	0

Abbreviations: IQR, interquartile range; PFC, pancreatic fluid collections; SMD, standardized mean difference.

All patients in both propensity‐matched cohorts achieved technical success (*n* = 45, *p* > 0.99). The median index procedure time was 43 min in the hPFC group compared to 42 min in the nhPFC group (*p* = 0.81). The median number of necrosectomies for patients with hemorrhagic PFCs was 1 (interquartile range [IQR] 1, 1.75). For non‐hemorrhagic collections, the median number of necrosectomies was 1.5 (IQR 1, 2). The *p*‐value comparing necrosectomies for hPFC and nhPFC was 0.41. The median duration of LAMS placement was similar between the two groups (26 vs. 36 days, hPFC vs. nhPFC, respectively, *p* = 0.62). The median length of hospitalization was 6 days in the hPFC group and 3 days in the nhPFC group (*p* = 0.25); however, more hPFC patients required ICU admission post‐procedure (33% vs. 17%, *p* = 0.26). The median ICU length of stay was 8 days for hPFC compared to 4 days for nhPFC (*p* = 0.40). The outcomes of patients are summarized in Tables 2 and 3.

**TABLE 2:** Comparative outcomes of patients with hemorrhagic pancreatic fluid collections and non‐hemorrhagic pancreatic fluid collections

**Patient characteristics**	**Hemorrhagic PFC (*n* = 15)**	**Non‐hemorrhagic PFC (*n* = 30)**	***p*‐Value**
Technical success, *n*, %	15, 100	30, 100%	>0.99
Clinical success, *n*, %	14, 93%	28, 93%	>0.99
Index procedure time (min), median (IQR)	43 (32, 62)	42 (28, 57)	0.81
LAMS duration (days), median (IQR)	26 (14, 53)	36 (19, 57)	0.62
Need for IR drainage, *n*, %	0, 0%	2, 7%	0.55
Surgical intervention, *n*, %	0, 0%	2, 7%	0.54
Hospital length of stay (days), median (IQR)	6 (3, 24)	3 (1, 14)	0.25
ICU admission, *n*, %	5 (33%)	5 (17%)	0.26
ICU length of stay (days), median (IQR)	8 (4, 8)	4 (3, 7)	0.40
In‐hospital mortality (*n*, %)	1, 7%	1, 3%	>0.99
30‐day hospital readmission rate (*n*, %)	5, 33%	2, 7%	0.032
Recurrence, *n*, %	0, 0	4, 13%	0.28
Adverse event, *n*, %	2, 13%	5, 17%	>0.99
Mortality at 3 months, *n*, %	2, 13%	1, 3%	0.25
Mortality at 6 months, *n*, %	4, 27%	2, 7%	0.09
Median length of follow‐up (months)	17.0	18.3	0.25

Abbreviations: ICU, intensive care unit; IR, interventional radiology; IQR, interquartile range; LAMS, lumen‐apposing metal stents; PFC, pancreatic fluid collections.

**TABLE 3:** Clinical outcomes of patients with hemorrhagic pancreatic fluid collection

**Patient no**.	**Age**	**Sex**	**BMI**	**Etiology of AP**	**Type of collection**	**Area of Collection (cm^2^)**	**Infection**	**CCI**	**PA**	**Gastric varices**	**DPDS**	**Procedure time (min)**	**Duration of LAMS placement (days)**	**Hospital LOS (days)**	**ICU admission post‐procedure**	**ICU LOS (days)**	**Readmission within 30 days**	**Readmission to ICU within 30 days**	**LAMS related AE**	**Mortality at 3 months**	**Mortality at 6 months**
1	56	F	22.32	ETOH	WON	159	Yes	1	No	No	No	33	53	8	No	–	No	No	No	No	No
2	33	M	24.59	ETOH	WON	153	Yes	2	No	No	No	31	50	6	No	–	No	No	No	No	No
3	63	F	25.68	GS	WON	65	Yes	4	No	No	No	69	63	25	Yes	8	Yes	Yes	No	Yes	Yes
4	67	F	32.61	GS	WON	45	Yes	5	No	No	Yes	63	35	90	Yes	29	Yes	No	No	No	No
5	61	M	26.25	UNK	WON	145	No	4	No	Yes	No	27	57	1	No	–	Yes	No	No	No	No
6	65	F	25.68	GS	WON	116	Yes	4	No	Yes	Yes	21	18	39	Yes	8	No	No	No	No	No
7	20	F	27.84	UNK	WON	55	No	3	No	No	Yes	32	14	3	No	–	No	No	No	No	No
8	43	M	30.52	ETOH	WON	42	No	1	No	Yes	No	47	70	1	No	–	No	No	No	No	No
9	79	F	20.49	HTG	WON	183	Yes	8	Yes, GDA	No	Yes	69	14	22	No	–	No	No	No	No	Yes
10	40	M	29.16	ETOH	WON	126	Yes	4	No	No	No	43	–	6	Yes	4	No	No	No	No	Yes
11	73	M	21.86	UNK	WON	117	No	4	No	No	Yes	62	14	3	No	–	No	No	No	No	No
12	43	M	23.17	ETOH	PC	27	No	1	Yes, Arc of Buhler	No	No	32	32	3	No	–	Yes	No	Yes, UGIB	No	No
13	71	M	23.72	GS	WON	120	Yes	10	No	No	Yes	51	20	24	Yes	3	Yes	Yes	Yes, UGIB	Yes	Yes
14	58	M	27.82	GS	WON	172	No	1	No	Yes	Yes	57	10	13	No	–	No	No	No	No	No
15	63	M	25.99	GS	WON	146	No	3	No	No	Yes	40	18	2	No	–	No	No	No	No	No

Abbreviations: AE, adverse event; AP, acute pancreatitis; BMI, body mass index; CCI, Charlson Comorbidity Index; DPDS, disconnected pancreatic duct syndrome; ETOH, alcohol; F, female; GDA, gastroduodenal artery; GS, gallstone; HTG, hypertriglyceridemia, ICU, intensive care unit; LAMS, lumen apposing metal stent; LOS, length of stay; M, male; PA, pseudoaneurysm; PC, pseudocyst, UGIB, upper gastrointestinal bleed; UNK, idiopathic; WON, walled‐off necrosis.

Clinical success was achieved in 14 (93%) hPFC patients and 28 nhPFC patients (*p* > 0.99). The single patient in the hPFC group without clinical success died prior to LAMS removal due to multiorgan failure secondary to infected walled‐off necrosis refractory to endoscopic drainage. This patient was not escalated to surgical step‐up due to poor candidacy in the setting of multiple comorbidities. Of the two clinical failures in the nhPFC group, one patient passed away from multiorgan failure despite dual‐modality drainage and escalation to open surgical necrosectomy. The other patient was successfully managed with surgical step‐up.

There were two LAMS‐related adverse events in the hPFC group. One patient developed self‐limiting upper gastrointestinal bleeding (UGIB) at the site of LAMS placement. The second patient developed UGIB after LAMS placement secondary to impingement of the stent on the apposing cyst wall causing bleeding and requiring epinephrine injection. In both patients, the LAMS were removed and replaced with double pigtail plastic stents. In the nhPFC group, there were five adverse events. Three patients had persistent fever following stent placement which was concerning inadequate drainage. On repeat endoscopy, necrotic material occluding the stent was seen. All three patients were treated with IV antibiotics and required endoscopic necrosectomy with the resolution of symptoms. One patient had an upper gastrointestinal bleed secondary to a pseudoaneurysm in the splenic artery requiring angioembolization. The last patient had postoperative abdominal pain due to smoldering pancreatitis requiring admission, intravenous fluids, and analgesics.

**FIGURE 4:**
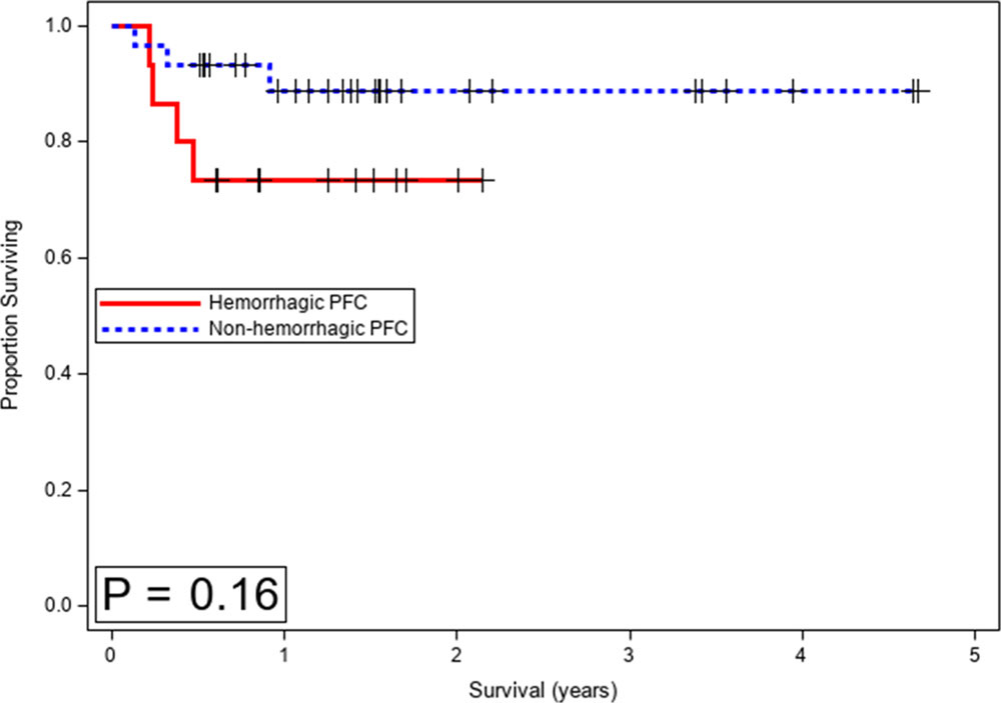
Kaplan Meier survival plot of propensity score matched cohorts comparing hemorrhagic and non‐hemorrhagic pancreatic fluid collections

Between the hPFC and nhPFC groups, the recurrence rate (0% vs. 13%, *p* = 0.28), the median length of follow‐up (17.0 vs. 18.3 months, *p* = 0.25), in‐hospital mortality (7% vs. 3%, *p* > 0.99), 3‐month mortality (13% vs. 3%, *p* = 0.25), and 6‐month mortality (27% vs. 7%, *p* = 0.09) did not reach statistical significance. However, when comparing post‐discharge 30‐day readmission rates, hPFC patients were more likely to be readmitted to the hospital (33% vs. 7%, *p* = 0.032). Intensive care unit readmission was seen in two patients in the hPFC group and none in nhPFC (*p* = 0.11). A comparison of adverse outcomes between hPFC and nhPFC collections is summarized in Table 4. The 1‐year survival estimate was 73.3% (standard error = 11.4) in the hPFC group and 88.9% (6.1) in the nhPFC group (*p* = 0.16) (Figure 4).

**TABLE 4:** Comparison of adverse outcomes between hemorrhagic and non‐hemorrhagic pancreatic fluid collections

**Hemorrhagic pancreatic fluid collections (*n* = 15)**	**Non‐hemorrhagic pancreatic fluid collections (*n* = 30)**
ICU admission (*n* = 5)	ICU admission (*n* = 5)
Septic shock (*n* = 3)	Septic shock (*n* = 2)
Acute respiratory distress syndrome (*n* = 1)	Acute respiratory distress syndrome (*n* = 2)
Cardiogenic Shock (*n* = 1)	Cardiogenic Shock (*n* = 1)
30‐day rehospitalization (*n* = 5, 33%)	30‐day rehospitalization (*n* = 2, 7%)
Pulmonary embolism (*n* = 1)	Postoperative abdominal pain (*n* = 1)
Sepsis (*n* = 1)	Sepsis (*n* = 1)
Upper GI bleed (*n* = 2)	
Hospital‐acquired pneumonia (*n* = 1)	
ICU readmission (*n* = 2)	ICU readmission (*n* = 0)
Upper gastrointestinal bleeding (*n* = 1)	
Hospital‐acquired pneumonia (*n* = 1)	
Mortality at 6 months (*n* = 4)	Mortality at 6 months (*n* = 2)
Clinical failure (*n* = 1)	Clinical failure (*n* = 1)
End‐stage COPD (*n* = 1)	Advanced heart failure (*n* = 1)
Advanced heart failure (*n* = 1)	
Pulmonary hemosiderosis (*n* = 1)	

Abbreviations: COPD, chronic obstructive pulmonary disease; ICU, intensive care unit; GI, gastrointestinal.

## DISCUSSION

hPFC result from autodigestion of blood vessels from pancreatic enzymes causing weakening of the vessel walls. These collections, which develop as a complication of pancreatitis, have an unclear influence on clinical outcomes. Recent advances in endoscopy have shown benefits to minimally invasive approaches in the management of PFC; however, extrapolation of these results to hemorrhagic collections is largely unknown.^9^ This study is the largest cohort of hPFC‐managed endoscopically and the first propensity‐matched analysis of hemorrhagic and non‐hemorrhagic collections. We address a paucity in the literature regarding the significance of intracystic hemorrhage in the management of PFC and hope to shed light on the importance of hPFC.

The underlying pathophysiology of acute pancreatitis complicated by bleeding is unique and distinctly different from acute pancreatitis.  Factors that contribute to worse outcomes associated with bleeding include coagulopathy, severe inflammatory state, severe protein deficiency, and systemic anticoagulation in patients with comorbidities such as cardiac arrhythmia. In addition, hemorrhagic PFC likely result in hemostatic dysequilibrium with procoagulant and antithrombotic factors contributing to anemia and poor clinical outcomes.  Our study demonstrates that endoscopists should be cognizant of intracystic hemorrhage as it likely yields worse outcomes. There was a statistically significant increase in 30‐day readmission rates amongst patients with hemorrhagic collections. In addition, patients in the hPFC group had a longer length of hospital stay, an increased likelihood of intensive care admission post‐procedure, and a higher 3‐ and 6‐month mortality; however, the analysis did not reach statistical significance. The clinical significance of these findings should not be understated. These results demonstrate that patients with hPFC are more likely to decline and have a more severe clinical course than their counterparts warranting close observation.

A standardized diagnostic and management algorithm is essential for the endoscopic management of hPFC. This includes CT angiography to assess the need for angioembolization of a pseudoaneurysm prior to endoscopic therapy. Endoscopic drainage of hPFC using LAMS provides a safe and effective alternative to surgical interventions, similar to non‐hemorrhagic PFC. However, due to the complexity of these collections and the risk of readmission, a multidisciplinary approach is warranted. Furthermore, patients may benefit from the close follow‐up to ensure the stability of the collection. This approach should promote improved outcomes, prevent patient morbidity, and ensure the safe resolution of the collection.

The existing literature on the management of intracystic hemorrhage is limited to a small collection of case series and case reports without long‐term follow‐up. Most of the reported cases in the literature have focused on surgical management of hPFC which has recently been supplanted by endoscopic and interventional radiology interventions due to their lower morbidity and mortality.^4^, ^10^, ^11^, ^12^, ^13^ Rana et al published a case series of 13 patients who underwent endoscopic transmural drainage of hPFC. All of these patients were diagnosed with hPFC based on the presence of high‐attenuated fluid on CT. Nine patients underwent transmural drainage with plastic stents and four with LAMS. All collections resolved after drainage and there were no complications reported. However, details of clinical outcomes including hospital length of stay, ICU admission, readmission rates, and follow‐up duration were not reported.^14^

The strengths of this study include its standardized approach to managing the subjects. In addition, all patients were managed by a multidisciplinary team with regular imaging obtained at the same institution. This eliminated the concern for variability in imaging quality. Furthermore, all patients were followed for at least 6 months with a gastroenterologist ensuring the capture of relevant clinical outcomes.

Our study is not without limitations. First and foremost, only 15 patients were identified during the study period to have hemorrhagic collections which limited the power of the analysis and likely underestimated the importance of hPFC. In addition, the presence of hemorrhage was identified by the endoscopist prior to drainage; therefore, treatment bias is a concern as this could have led to a more conservative management approach. In addition, all procedures were performed upon consultation with a multidisciplinary team including advanced therapeutic endoscopists, a hepatobiliary surgeon, and abdominal radiologists at a large tertiary care facility. This may prevent the extrapolation of these findings to all institutions.

In conclusion, our findings show that hPFC warrant close attention due to their impact on clinical outcomes. Larger studies are indicated to examine the significance of hPFC.

## CONFLICT OF INTEREST

Dr Rishi Pawa is a consultant for Boston Scientific. Dr. Girish Mishra is a consultant for Cook Endoscopy and Pentax Medical.

## References

[1] Dorrell R , Pawa S , Pawa R . Endoscopic management of pancreatic fluid collections. J Clin Med 2021; 10: 284. 33466752 10.3390/jcm10020284 PMC7835868

[2] Van Santvoort H , Besselink M , Cirkel G , Gooszen H . A nationwide Dutch study into the optimal treatment of patients with infected necrotising pancreatitis: The PANTER trial. Ned Tijdsch Geneeskund. 2006; 150: 1844–6. 16967597

[3] van Santvoort HC , Bakker OJ , Bollen TL *et al.* A conservative and minimally invasive approach to necrotizing pancreatitis improves outcome. Gastroenterology 2011; 141: 1254–63. 21741922 10.1053/j.gastro.2011.06.073

[4] Balthazar EJ , Fisher LA . Hemorrhagic complications of pancreatitis: Radiologic evaluation with emphasis on CT imaging. Pancreatology 2001; 1: 306–13. 12120209 10.1159/000055829

[5] Kudaravalli P , Garg N , Pendela VS , Gambhir HS . Hemorrhagic pancreatic pseudocyst: A rare complication. Am J Emerg Med 2021; 43: 243–4. 32197717 10.1016/j.ajem.2020.03.020

[6] Hashimoto BE , Laing FC , Jeffrey RB Jr. , Federle MP . Hemorrhagic pancreatic fluid collections examined by ultrasound. Radiology 1984; 150: 803–8. 6695082 10.1148/radiology.150.3.6695082

[7] Charlson ME , Pompei P , Ales KL , MacKenzie CR . A new method of classifying prognostic comorbidity in longitudinal studies: Development and validation. J Chronic Dis 1987; 40: 373–83. 3558716 10.1016/0021-9681(87)90171-8

[8] Cotton PB , Eisen GM , Aabakken L *et al.* A lexicon for endoscopic adverse events: Report of an ASGE workshop. Gastrointest Endoscopy 2010; 71: 446–54. 10.1016/j.gie.2009.10.027 20189503

[9] van Brunschot S , van Grinsven J , van Santvoort HC *et al.* Endoscopic or surgical step‐up approach for infected necrotising pancreatitis: A multicentre randomised trial. Lancet 2018; 391: 51–8. 29108721 10.1016/S0140-6736(17)32404-2

[10] Okamura K , Ohara M , Kaneko T *et al.* Pancreatic pseudocyst ruptured due to acute intracystic hemorrhage. Case Rep Gastroenterol 2017; 11: 755–62. 29430229 10.1159/000485236 PMC5803717

[11] Masatsugu T , Yamaguchi K , Yokohata K , Mizumoto K , Chijiiwa K , Tanaka M . Hemorrhagic pseudocyst and pseudocyst with pseudoaneurysm successfully treated by pancreatectomy: Report of three cases. J Hepatobiliary Pancreat Surg 2000; 7: 432–7. 11180866 10.1007/s005340070040

[12] Parra V , Boumitri C , Kedia P , Sharaiha RZ , Kahaleh M . Hemorrhagic pancreatic necrosis drainage by using an esophageal stent. Gastrointest Endosc 2015; 81: 458–9. 25038003 10.1016/j.gie.2014.05.325

[13] Huang YT , Liu Q , Zhang BS . Long‐term results of surgical treatment for acute hemorrhagic necrotizing pancreatitis. Chin Med J 1993; 106: 500–3. 8243120

[14] Rana SS , Sharma R , Gupta R . EUS‐guided transmural drainage of hemorrhagic pancreatic fluid collections without associated arterial pseudoaneurysms. Endosc Ultrasound 2021; 10: 396–7. 34494588 10.4103/EUS-D-21-00045 PMC8544005

